# Supplementary material for: Identification of Subject-Specific Immunoglobulin Alleles From Expressed Repertoire Sequencing Data
Source: Front Immunol. 2019 Feb 13;10:129. doi: 10.3389/fimmu.2019.00129 (PMC6381938; doi:10.3389/fimmu.2019.00129)
Supplement: Supplementary file 1 [file Data_Sheet_1.pdf]

## SUPPLEMENTRY FIGURES

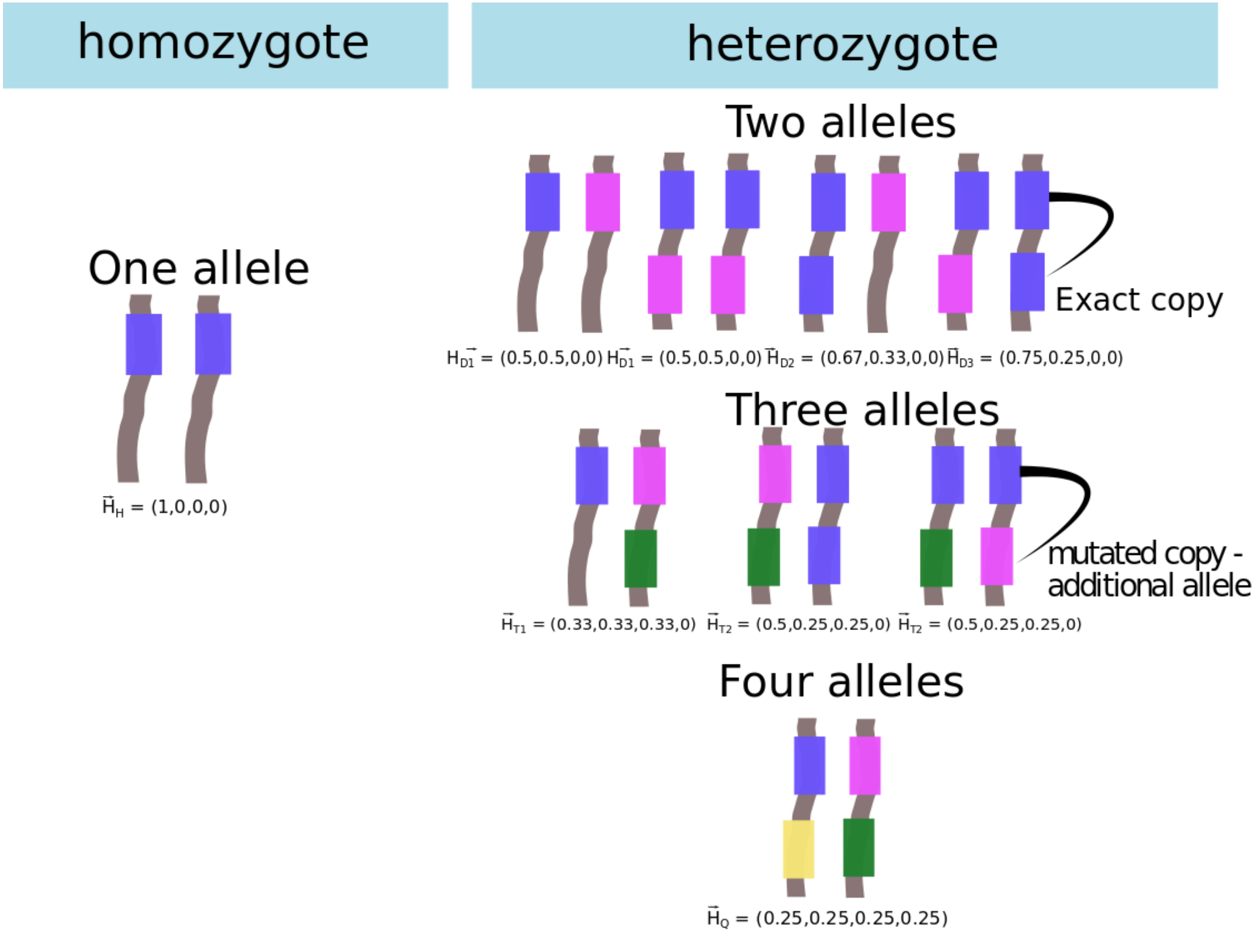


**Figure S1: Models for genotype calls considered by the Bayesian method.**

The Bayesian genotyping method considers four potential models to explain the expression patterns of each gene. In the homozygote model (left), the same allele is carried on both chromosomes. In the heterozygote model (right), several possibilities are considered: in addition to two different alleles, one on each chromosome, up to two repeats of each allele on each chromosome were considered. Assuming polymorphisms can be present in each repeat, this results in up to four different alleles carried by a single individual. Below each possibility, the corresponding allele probability distribution ($\vec{H}$) is listed.

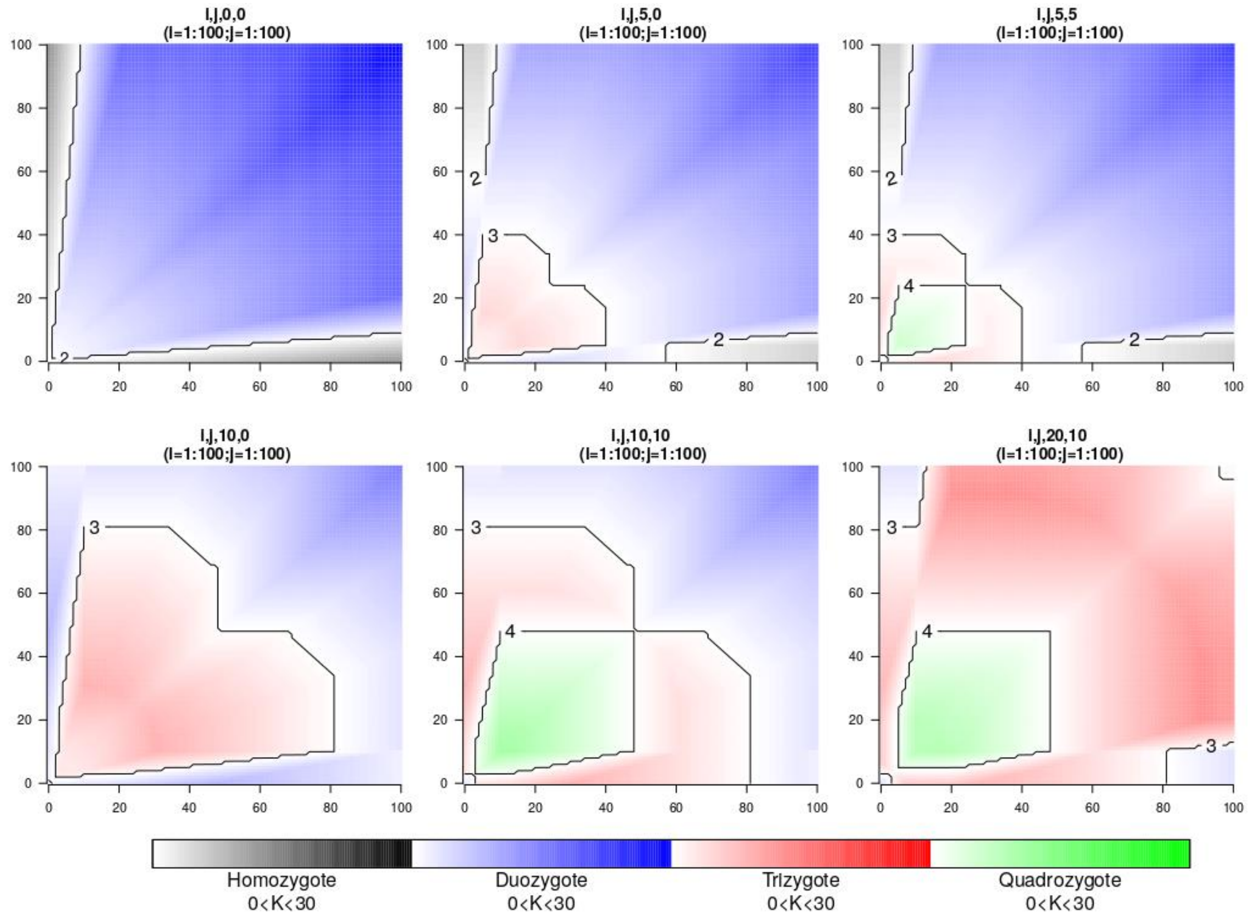


**Figure S2: Genotype call and certainty levels as reflected by allele sequence counts for a single gene.**

The counts for the top two alleles in terms of frequency are represented by the Y and X axes, while counts for the third and fourth alleles are set for each panel and appear on the panel title. For example, for i,j,10,5 the counts of the third and fourth alleles are 10 and 5, respectively. The color reflects the genotype call: homozygote (black), douzygote - heterozygote with two alleles (blue), trizygote - heterozygote with three alleles (red) and quadrozygote - heterozygote with four different alleles (green). The intensity of the colors reflects the certainty level (K) of each genotype call.

## SUPPLEMENTRY TABLES

| Data Set | Gene | Allele(s) | Unmutated Sequence Count (Allele) | Unmutated Sequence Count (Gene) | AA change – replacement(R) /silent(S) |
|---|---|---|---|---|---|
| **MK08** | IGHV1-18 | 01_A190G | 29 | 77 | R |
| **AR05** | IGHV1-18 | 01_A196G | 276 | 566 | R |
| **M2** | IGHV1-18 | 01_T111C | 732 | 1849 | S |
| **M4** | IGHV1-2 | 02_T163C | 96 | 227 | R |
| **M5** | IGHV1-2 | 02_T163C | 276 | 281 | R |
| **hu420143** | IGHV1-2 | 02_T163C | 819 | 2028 | R |
| **HD13** | IGHV1-2 | 02_T163C | 58 | 100 | R |
| **AR05** | IGHV1-2 | 02_T163C | 312 | 682 | R |
| **MK04** | IGHV1-2 | 02_T163C | 60 | 94 | R |
| **TW071A** | IGHV1-69 | 01_G54A | 65 | 106 | S |
| **MK08** | IGHV1-69 | 04_G48A_A163G_A244G | 38 | 111 | S,R,R |
| **M3** | IGHV1-69 | 06_C191T | 103 | 268 | R |
| **MK06** | IGHV1-69 | 06_C191T | 94 | 468 | R |
| **MK05** | IGHV1-69 | 12_G48A | 44 | 250 | S |
| **PGP1** | IGHV1-8 | 02_G234T | 1548 | 3500 | S |
| **MK05** | IGHV1-8 | 02_G234T | 91 | 91 | S |
| **PGP1** | IGHV2-70 | 01_T164G | 221 | 350 | R |
| **M2** | IGHV3-11 | 03_C300T | 420 | 719 | S |
| **420IV** | IGHV3-11 | 03_T13G | 1625 | 2690 | R |
| **PGP1** | IGHV3-20 | 01_C307T | 361 | 424 | R |
| **MK05** | IGHV3-20 | 01_C307T | 61 | 61 | R |
| **HD10** | IGHV3-30 | 18_C75G | 42 | 116 | S |
| **420IV** | IGHV3-43 | 01_A112G_C222T_A286G | 175 | 408 | R,S,R |
| **MK05** | IGHV3-48 | 01_A39C | 79 | 133 | S |
| **M2** | IGHV3-64 | 05_A210C_G265C | 98 | 104 | S,R |
| **MK05** | IGHV4-30-4 | 01_T120C | 33 | 33 | S |
| **TW070B** | IGHV4-34 | 01_A220G | 47 | 412 | R |
| **TW071A** | IGHV4-34 | 01_A220G | 78 | 910 | R |
| **TW071B** | IGHV4-34 | 01_A220G | 74 | 766 | R |
| **TW071A** | IGHV4-34 | 01_A220G_A225G | 58 | 910 | R,S |
| **TW059A** | IGHV4-39 | 01_A291G | 21 | 182 | S |
| **TW071A** | IGHV4-39 | 01_A291G | 12 | 111 | S |
| **TW070B** | IGHV4-39 | 07_A220G_A225G | 43 | 258 | R,S |
| **TW064A** | IGHV4-39 | 07_C288A | 31 | 81 | S |
| **TW070A** | IGHV4-39 | 07_C300T | 19 | 109 | S |
| **TW070B** | IGHV4-39 | 07_C300T | 26 | 258 | S |
| **TW064A** | IGHV4-59 | 01_T288C | 21 | 85 | S |
| **TW064B** | IGHV4-59 | 01_T288C | 20 | 76 | S |
| **TW046A** | IGHV4-59 | 01_T288C | 38 | 62 | S |
| **TW059A** | IGHV4-59 | 01_T288C | 28 | 67 | S |
| **TW059B** | IGHV4-59 | 01_T288C | 26 | 62 | S |
| **TW070A** | IGHV4-59 | 01_T288C | 34 | 74 | S |

| | | | | | |
|---|---|---|---|---|---|
| **TW070B** | IGHV4-59 | 01_T288C | 46 | 120 | S |
| **TW071A** | IGHV4-59 | 01_T288C | 13 | 57 | S |
| **TW059A** | IGHV4-59 | 08_A291G | 16 | 67 | S |
| **TW059B** | IGHV4-59 | 08_A291G | 9 | 62 | S |
| **TW070B** | IGHV4-59 | 08_A291G | 13 | 120 | S |
| **MK02** | IGHV4-61 | 02_A234G | 27 | 27 | R |

**Table S1**: **Novel Immunoglobulin IGHV alleles discovered by TIgGER.**

For each biological sample in which a novel allele was discovered by TIgGER and determined to be in the genotype, the gene, nearest IgGRdb allele, and the polymorphisms that differentiate the novel allele from it are listed. (e.g., IGHV1-2 allele 02_T163C represents that the novel allele is most similar to IGHV1-2*02 and contains a T → C polymorphism at position 163.) The fourth and fifth columns indicate the number of unique sequences from the dataset that represent unmutated examples of the novel allele and the number of unique sequences from the dataset that represent unmutated examples of any allele of the given gene that was included in the genotype. The rightmost column indicates if the polymorphisms resulted in amino acid (AA) changes relative to the nearest IgGRdb allele, which is listed in the third column.

| Gene Target | Forward Primer (5'-3') | Reverse Primer (5'-3') |
|---|---|---|
| *IGHV1-2* | GAGACTCTGTCACAAACAAACCA | GTGTGTTCTCTTTCTCATCTTGGA |
| *IGHV1-8* | CCACTGTGAATTCGGCTTCT | CTGTGAAATACCCTGCCTCA |
| *IGHV1-69* | GCACCTTCATGGAATGTTTG | TGAAATACCCTGCCTCATGC |
| *IGHV3-20* | GACGTTTGTGTCTGGGCTCT | CTGCAGCTCTGGGAGAGG |

**Table S2**: **Primers used to fully amplify the exons and introns of each target IGHV gene locus.**

| Gene | Alleles | Counts | Total | $K_H$ | $K_D$ | $K_T$ | $K_Q$ | K |
|---|---|---|---|---|---|---|---|---|
| IGHV1-2 | 02,04 | 479,198 | 677 | -220.75 | -6.32 | -100.15 | -222.88 | 93.83 |
| IGHV1-3 | 01,02 | 136,4 | 140 | 3.85 | -19.49 | -43.18 | -70.65 | 23.33 |
| IGHV1-8 | 01,03 | 307,261 | 568 | -1000.00 | 0.33 | -67.39 | -165.00 | 67.71 |
| IGHV1-18 | 01,04 | 598,1 | 599 | 1.50 | -127.85 | -231.09 | -1000.00 | 129.34 |
| IGHV1-24 | 01 | 81 | 81 | 4.73 | -13.01 | -26.99 | -43.01 | 17.74 |
| IGHV1-46 | 01 | 523 | 523 | 1.51 | -113.02 | -203.26 | -306.72 | 114.53 |
| IGHV1-58 | 01,02 | 21,8 | 29 | -5.30 | 3.01 | -1.07 | -6.35 | 4.08 |
| IGHV1-69 | 01,04,06,02 | 334,295,209,1 | 839 | -1000.00 | -218.28 | 3.16 | -103.18 | 106.35 |

**Table S3:** Certainly values for different genotypes calculated by the Bayesian inference method for subject PGP1, along with the overall Bayes factor (K). Results are shown for a subset of genes.
